# Proteome effects of genome-wide single gene perturbations

**DOI:** 10.1038/s41467-022-33814-8

**Published:** 2022-10-18

**Authors:** Merve Öztürk, Anja Freiwald, Jasmin Cartano, Ramona Schmitt, Mario Dejung, Katja Luck, Bassem Al-Sady, Sigurd Braun, Michal Levin, Falk Butter

**Affiliations:** 1 Institute of Molecular Biology (IMB), Mainz, Germany; 2 Department of Microbiology & Immunology, George Williams Hooper Foundation, University of California San Francisco, San Francisco, CA USA; 3 Department of Physiological Chemistry, Biomedical Center, Ludwig-Maximilians University of Munich, Planegg-Martinsried, Germany; 4 Institute for Genetics, Justus-Liebig-University Giessen, Giessen, Germany

**Keywords:** Fungal systems biology, Regulatory networks, Proteomics, Gene expression

## Abstract

Protein abundance is controlled at the transcriptional, translational and post-translational levels, and its regulatory principles are starting to emerge. Investigating these principles requires large-scale proteomics data and cannot just be done with transcriptional outcomes that are commonly used as a proxy for protein abundance. Here, we determine proteome changes resulting from the individual knockout of 3308 nonessential genes in the yeast *Schizosaccharomyces pombe*. We use similarity clustering of global proteome changes to infer gene functionality that can be extended to other species, such as humans or baker’s yeast. Furthermore, we analyze a selected set of deletion mutants by paired transcriptome and proteome measurements and show that upregulation of proteins under stable transcript expression utilizes optimal codons.

## Abstract (web summary)

Protein abundance is controlled at the transcriptional, translational and posttranslational levels. Here, Öztürk et al. determine proteome changes resulting from individual knockout of 3308 nonessential genes in the yeast *S. pombe*, infer gene functionality, and show that protein upregulation under stable transcript expression utilizes optimal codons.

## Introduction

Our understanding of global gene regulation has been advanced by large-scale investigations^1^. While transcriptomics studies are easily scalable to thousands of samples^2^, recent large-scale proteome studies were restricted to a few hundred conditions^3–6^. Single-gene knockout strains in baker’s yeast have been systematically analyzed in larger experiments with 1484 transcriptomes^2^ or 174 proteomes^6^ to understand principles of global gene regulation. The popularity of *S. cerevisiae* in these studies is likely connected to easier generation of gene knockouts and an early availability of the gene knockout collection^7^. However, certain features of *Schizosaccharomyces pombe*, such as splicing, RNA interference, chromosome structure, cytokinesis, and mitochondrial metabolism, are more similar to those of humans than to those of baker’s yeast^8,9^, suggesting that it is an optimal model organism for studying gene regulatory principles in eukaryotes. *S. pombe* is already a well-established model organism^10^ also for omics studies with several transcriptomics^11–13^ and proteomics datasets^14,15^, two genome-wide genetic interactions^16,17^ and a large yeast-two-hybrid (Y2H) study^12^.

Despite being technically challenging, larger proteomics studies are instrumental to further deepen our understanding of post-transcriptional control, i.e., protein expression changes that are not predetermined by mRNA levels. Indeed, many studies report pronounced differences between the transcriptome and proteome, as reviewed in Buccitelli & Selbach^1^, emphasizing the importance of measuring protein levels rather than just mRNA. Here, we provide the systematic proteome analysis of 3308 single-gene knockouts in *S. pombe*.

## Results and discussion

### Proteomics screen with thousands of single gene deletion strains

**Fig. 1:**
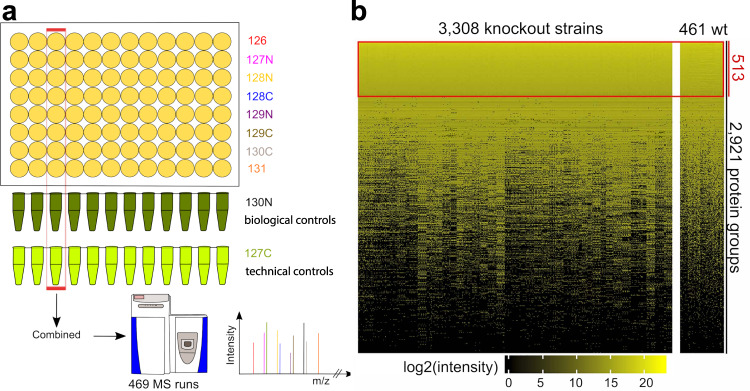
Proteomics screen to study proteome expression in a knockout library. **a** Schematics of the experimental design for quantifying protein expression levels in the *S. pombe* knockout collection by quantitative proteomics using 10plex TMT. Each MS run contained a technical (127 C label) and a biological control (130 N label), while the biological control samples were grown along with the knockout samples. **b** Normalized protein expression levels of 3308 knockout strains and 461 wild-type replicates. The complete dataset contains quantification of 2921 protein-coding genes, of which 513 proteins were quantified across all samples. Source data are provided as a Source Data file.

To systematically investigate the effect of individual gene deletions on the proteome, we examined the *S. pombe* haploid knockout collection with 3308 individually deleted genes^18^. We took advantage of the 96-well array format of the deletion library (Bioneer, version 3.0) by combining eight knockout strains with 2 different controls per mass spectrometry (MS) measurement and assessed the proteome of the 3308 knockout strains by quantitative proteomics using 10plex TMT (tandem mass tag)^19^ (Fig. 1a). One of the controls served as the technical control required for run-to-run normalization and was generated by growing a large batch of *S. pombe* wild-type (WT) cells. The other biological controls consisted of *S. pombe* wild-type replicates grown alongside the knockout strains to be able to judge biological protein expression level variation in wild-type cells.

Overall, we quantified 2921 proteins (56% of protein-coding genes), with a mean of 1596 proteins per strain, ranging from 1369 to 1996 proteins (Fig. 1b). Quality control showed that data quality is high, as the number of MS and MS/MS spectra as well as the number of identified peptides and proteins is comparable among runs (Supplementary Fig. 1a–e). Furthermore, we determined labeling efficiency (~98%) and observed very few incidences of overlabeling (~6%) (Supplementary Fig. 1f). As expected, there was a general tendency of highly abundant proteins to be quantified more often in the dataset (Supplementary Fig. 1g). Of the 2921 quantified proteins, 985 (34%) could be quantified in at least 90% of the knockout strains and 1548 (53%) in at least 50% of the knockout strains.

Cross-run normalization is known to be one of the key challenges in TMT experiments^20^. We noted that strains grown in the same 96-well plate exhibited higher correlations than strains grown in different 96-well plates (Supplementary Fig. 1h). Even more striking, samples from the same plate were more similar when they were measured in the same MS run (Supplementary Fig. 1h). To assess the tendencies and variability of individual measurements for quality control and data normalization, we focused on the 513 proteins that were identified across all 3769 samples (3308 knockout and 461 wild-type strains) (Fig. 1b). We used our technical control to normalize quantitation values, which already reduced the observed batch effects (Supplementary Fig. 1i, “WT technical”). To correct for the remaining batch effect, we explored several available strategies (Supplementary Fig. 1i) and applied an adjusted median method that assumed an identical average intensity for each protein among the different MS measurements. When compared to other established methods, the performance of this approach showed optimal results with a low bias coefficient and a high median of the Pearson’s correlation coefficient to the original data (Supplementary Fig. 1j). Our normalized data now allow extensive investigation of the effect of thousands of individual single-gene knockouts on proteome homeostasis.

### Certain gene categories show extensive proteome remodeling upon knockout

**Fig. 2:**
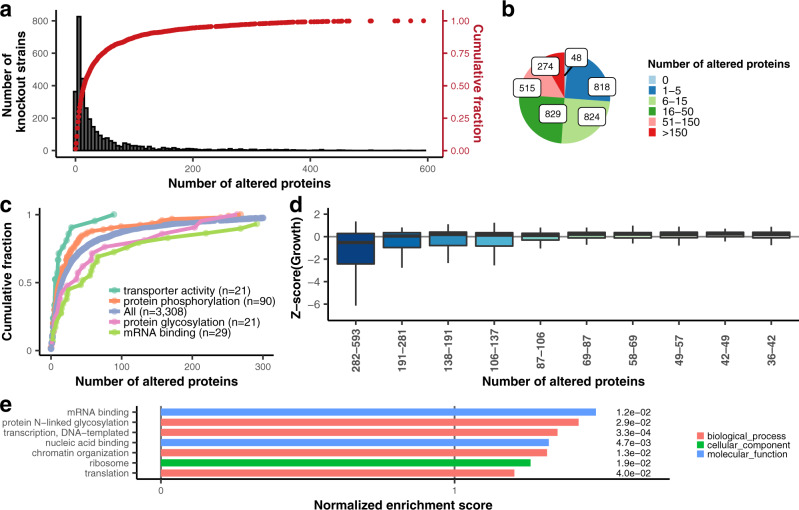
Knockout strains with high proteome remodeling tend to have reduced fitness and are involved in gene regulation. **a** Histogram (black) and cumulative scatterplot (red) showing the distribution of the number of altered proteins per strain within the knockout library. **b** Pie chart of altered proteins per knockout strain. **c** Analysis of altered proteins for different classes. **d** Growth behavior binned by the observed number of altered proteins in 33 fractions across all strains illustrating a significant growth deficiency in strains with extensive proteome remodeling. The first 10 fractions, each containing 100 strains, are shown here. The interquartile range (IQR) is depicted by the box with the median represented by the center line. Whiskers maximally extend to 1.5*IQR. **e** Selected gene ontology terms of the gene set enrichment analysis performed for the knockout strains ranked by the number of altered proteins. Permutation based *p*-values are shown to the right of each bar. Source data are provided as a Source Data file.

We determined the altered number of proteins per knockout strain by comparing their expression level to all 461 wild-type replicates (Fig. 2a). We considered proteins with two-tailed z-test FDR (Benjamini–Hochberg corrected *p*-values) less than 0.05 to be differentially expressed (see methods). We observed that only 0.02% (48) of knockout strains showed no difference compared to the proteome of wild-type cells, and approximately 4.2% (138) showed a single differentially expressed protein (Fig. 2b). On average, 45.6 proteins per strain had altered abundance, while only 8.3% (274) of strains have more than 150 differentially expressed proteins (Fig. 2b). The number of changed proteins varies between different pathways; for example, in mRNA binding and in protein glycosylation, on average, more proteins change, while for protein phosphorylation and transporter proteins, we observe less changes in protein expression when compared to the average of all knockouts (Fig. 2c). For further analyses, we provide the full information, including statistical measures, for each individual strain and each altered protein in our *S. pombe* ProtQuant database (www.butterlab.org/SpProtQuant).

Changes in protein homeostasis could be a direct consequence of the lack of protein or indirectly caused by pleiotropic effects and reduced fitness. To determine whether extensive proteome remodeling is associated with growth disadvantages, we divided the 3308 knockout strains into 33 bins according to the number of altered proteins. The first bin contained the 100 strains with the most extensive proteome remodeling (more than 282 proteins with changed expression per knockout compared to wild-type). Matching these data with growth measurements we performed for each knockout strain, we show that strains in the first bin exhibited significantly slower growth (Kruskal–Wallis, *p* < 2.2e^−16^) than all other knockout strains (Fig. 2d and Supplementary Fig. 2a). Gene set enrichment analysis (GSEA) of the knockout strains ranked by the number of altered proteins showed overrepresentation of terms related to the regulation of gene expression, RNA metabolism and protein metabolism for strains with higher proteome remodeling (Fig. 2e). Notably, genes belonging to these gene ontology (GO) categories were also enriched among essential genes (Supplementary Fig. 2b). This may suggest that extensive expression remodulation of the cell at the proteome level is attempted at the cost of fitness to ensure survival. In the case of essential genes, this is not achievable and thus results in cell death.

### Co-expression patterns and strain similarity reveal complex and pathway associations

It has been shown that proteins belonging to the same complex or pathway are often co-expressed^21^. Thus, correlative expression comparison can be used to infer functional information for currently uncharacterized proteins. To benchmark co-expressed groups of proteins across the knockout strains from our genome-wide proteome analysis, we checked whether pairwise protein correlations agree with previously reported pathways, complexes and transcriptome co-expression patterns using STRINGdb^22^. Indeed, there is good agreement between STRINGdb associations and higher Pearson correlation coefficients of protein co-expression, demonstrating that proteome co-expression from our screen recapitulates STRINGdb associations (Supplementary Fig. 3a).

As the overall correlations appear meaningful, we next inferred the function of individual proteins by using the functional information from co-expressed proteins, similar to what is commonly done at the transcript level. We used the supersom clustering algorithm^23^ based on self-organizing maps (SOM) to group proteins based on their abundance tendencies across all knockout strains. After quality assessment of the initial 144 clusters, we kept 113 high-confidence clusters with low mean distances to the center of the respective cluster (<75 percentile of all distances). These clusters contained 1171 proteins with high co-expression correlations between protein pairs belonging to the same cluster (Supplementary Fig. 3b–e). To benchmark these clusters, we attempted to use a previously published Y2H interaction screen^24^, but the overlap was too small (Supplementary Fig. 3f). However, when we used reported *S. pombe* genetic interactions^16^, we found significantly higher genetic interaction profile correlations for proteins that belong to the same cluster (Supplementary Fig. 3g). Importantly, these clusters showed twice as high STRINGdb connectivity scores than expected by chance (Supplementary Fig. 3h, i).

**Fig. 3:**
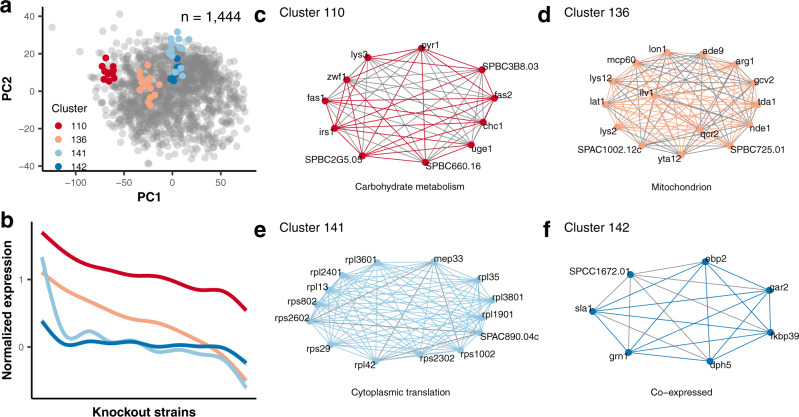
Protein co-expression clusters determined by neural network-based clustering allow improved annotation. **a** Principal component plot of the 1444 protein groups quantified in at least 1894 knockout strains (57% of all knockout strains). Principle component analysis (PCA) was performed based on the z-score transformed abundances of the proteins across the knockout strains. Four selected self-organizing map (SOM) clusters are highlighted in different colors and contain the proteins named in the network plots **c**–**f**. **b** Z-scored average intensity profiles of the selected clusters outlining the changes in their expression behavior across knockout strains**. c**–**f** The selected 4 clusters are examples enriched for specific cellular pathways or subcellular localization as assigned by the STRING database (colored edges); newly identified associations are depicted with gray edges. Nodes depict individual proteins. Source data are provided as a Source Data file.

To functionally annotate these clusters, we employed previously defined STRINGdb interactions and available GO annotations (Fig. 3). Here, we recapitulated known subcellular localizations and extended existing annotations. For instance, all proteins in cluster 136 (Fig. 3d) are annotated mitochondrial proteins in *S. pombe*. In cluster 141 (Fig. 3e), we observed clustering of 12 known ribosomal proteins, and according to pombase 19, one predicted ribosomal protein (SPAC890.04c) and a biogenesis factor (Mep33) for the ribosome. This is also applicable for biological pathways, as 6 of 7 proteins of cluster 142 (Fig. 3f) were previously described to be co-expressed on mRNA level, and in fact, they are all involved in translational regulation. Overall, we recapitulated 1487 pairwise associations included in the STRINGdb and provided 5025 putative new associations based on our clustering. Overall, we added annotations for 1150 proteins in *S. pombe* by this strategy.

As our study encompasses a genome-wide proteomic analysis of the full deletion library, we also investigated which of the deletion strains exhibit similar alterations in their proteome. The knockout library contained only nonessential genes for which there is less information available in the STRINGdb^22^ and Reactome^25^ databases compared to essential genes (Supplementary Fig. 4a–c). To improve functional annotation of even more nonessential genes, we applied the same SOM approach in the orthogonal way to cluster our 3308 knockout strains together with all 461 wild-type measurements by similarity of their proteome alteration, which resulted in 121 clusters. Notably, clusters predominantly contained either wild-type or knockout strains (Supplementary Fig. 4d). Confirming the clustering approach, we observed better correlation within the clusters than by pairwise comparison between strains from different clusters (Supplementary Fig. 4e–h). Due to insufficient information in the STRINGdb and the Reactome databases, we instead used enriched GO annotation of altered proteins within a cluster of knockout strains to suggest the functionality of their respective cluster. Using this approach, we were able to extend annotations of 2177 nonessential genes and recapitulate 16% of the mostly homology-inferred annotations (68% are inferred) but also increase the number of annotations by seven times using our proteome data from *S. pombe*.

In summary, by analyzing our genome-wide deletion library proteomes in both dimensions - changes in protein abundances across knockout strains and changes in strains across the entire proteome—we demonstrated that the information in our dataset is able to suggest functional annotations for clusters containing 2408 proteins. All protein clusters and the respective annotations can be explored via our *S. pombe* ProtQuant database (www.butterlab.org/SpProtQuant).

### Quantitative strainwise transcriptome-proteome comparison uncovers post-transcriptional regulation

**Fig. 4:**
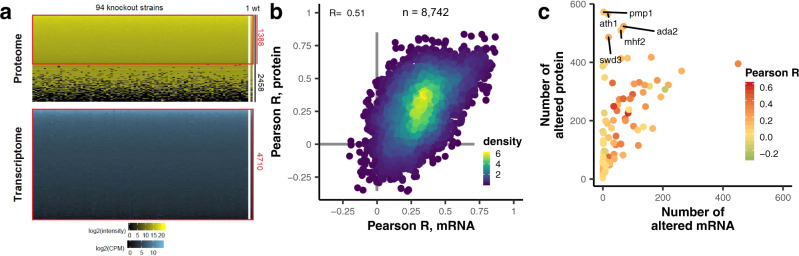
Correlation of transcriptome and proteome for 94 selected knockout strains reveals post-transcriptional regulators. **a** Heatmaps of proteome expression (yellow-black) and transcriptome expression (blue-black) obtained by measuring the same samples by quantitative proteomics and RNA-Seq (*n* = 4). **b** Scatterplot of the pairwise Pearson’s correlation coefficients between all possible knockout strain pairs (*n* = 8742) at the transcriptome versus proteome levels shows moderate correlation (R = 0.51). **c** Scatterplot comparing the number of altered proteins and the number of altered transcripts for all 94 strains. The color scale shows the Pearson correlation coefficient between the transcriptome and proteome of the respective knockout strain. Source data are provided as a Source Data file.

We selected 94 knockout strains across the full spectrum of the number of altered proteins (Supplementary Fig. 5a). The 94 genes deleted in these individual strains belong to a diversity of processes, such as transcription, the cell cycle and redox processes, with 20 proteins featuring the GO term ‘cytoplasmic translation’ being the largest group (Supplementary Fig. 5b). To discriminate between transcriptomic and proteomic effects of the gene knockout, we performed transcriptome (Supplementary Fig. 6) and proteome (Supplementary Fig. 7) measurements for these 94 genes in quadruplicates, resulting in data for 4710 transcripts and 2458 proteins (Fig. 4a). Notably, while replicates in general increase precision, comparing the 94 remeasured proteomes with replicates to the original screen shows a good correlation of protein expression values (Pearson’s R 0.7–0.9) (Supplementary Fig. 5c), validating the high reproducibility and precision of the quantitation method.

Having paired transcriptome and proteome data, we compared the strains on either transcriptome or proteome using the similarity of their expression changes in contrast to wild-type (Supplementary Fig. 5d). Correlations between strains at the transcriptome level were different from correlations among the strains at the proteome level (R = 0.51), suggesting posttranslational regulation in some of these strains (Fig. 4b). This should be acknowledged, as mRNA is commonly used as a proxy for protein levels in gene expression studies^26^.

To identify knockout strains with posttranscriptional effects, we performed correlation analysis between the transcriptome and proteome in each strain (Supplementary Fig. 5e, f). For the 94 selected strains, this correlation varied between −0.2 and 0.6, demonstrating that this is strongly dependent on the individual gene knockout. For example, our paired transcriptome/proteome data showed that pmp1Δ, ath1Δ, ada2Δ, mhf2Δ, and swd3Δ exhibit a high number of differentially expressed proteins, while they have a rather small number of differentially expressed mRNAs (Fig. 4c).

### Protein upregulation at stable mRNA levels is linked to optimal codon usage

Having 94 paired transcriptome/proteome datasets lent themselves to identifying genes under posttranscriptional regulation in multiple conditions. To this end, we analyzed the transcriptome/proteome correlation of the 1706 measured gene products across the knockout strains. We observed varying correlations for individual transcript/protein pairs for these genes, ranging from anti-correlation (R = −0.84) to near perfect correlation (R = 0.98) (Supplementary Fig. 8a). Among these genes, 750 had significantly high transcript/protein correlations (*p* < 0.05), and 90 of them had statistically significant anti-correlations (*p* < 0.05), i.e., protein upregulation can be predicted from mRNA downregulation and vice versa (Supplementary Fig. 8b). While for the highly correlated pairs, there were no strongly enriched GO terms, for the anticorrelated pairs, we found enrichment of GO terms related to RNA processing and protein phosphorylation (Supplementary Fig. 8c, d). However, the noted anticorrelation can stem from the low expression levels of the proteins that are thus more difficult to precisely quantify and should be interpreted cautiously (Supplementary Fig. 8e, f).

Next, we focused on changes in protein expression abundance under stable mRNA levels to investigate post-transcriptional features more directly. To this end, we clustered the subset of genes with protein expression changes under stable mRNA levels (*n* = 769) into two groups using PAM (partitioning around medoids) clustering based on their regulation at the protein level (Supplementary Fig. 9a). Looking into the differential regulation dynamics of the two clusters, we found that the first group contains genes that tend to be upregulated at the proteome level across most of the strains, while the second group contains genes that tend to be generally downregulated (Supplementary Fig. 9b). Gene ontology analysis of these two groups revealed various cellular metabolic process-related terms, especially amino acid biosynthesis, being enriched among upregulated genes and depleted in the downregulated genes (Supplementary Fig. 9c). These findings reveal that some processes related to amino acid metabolism might be mainly under translational control.

**Fig. 5:**
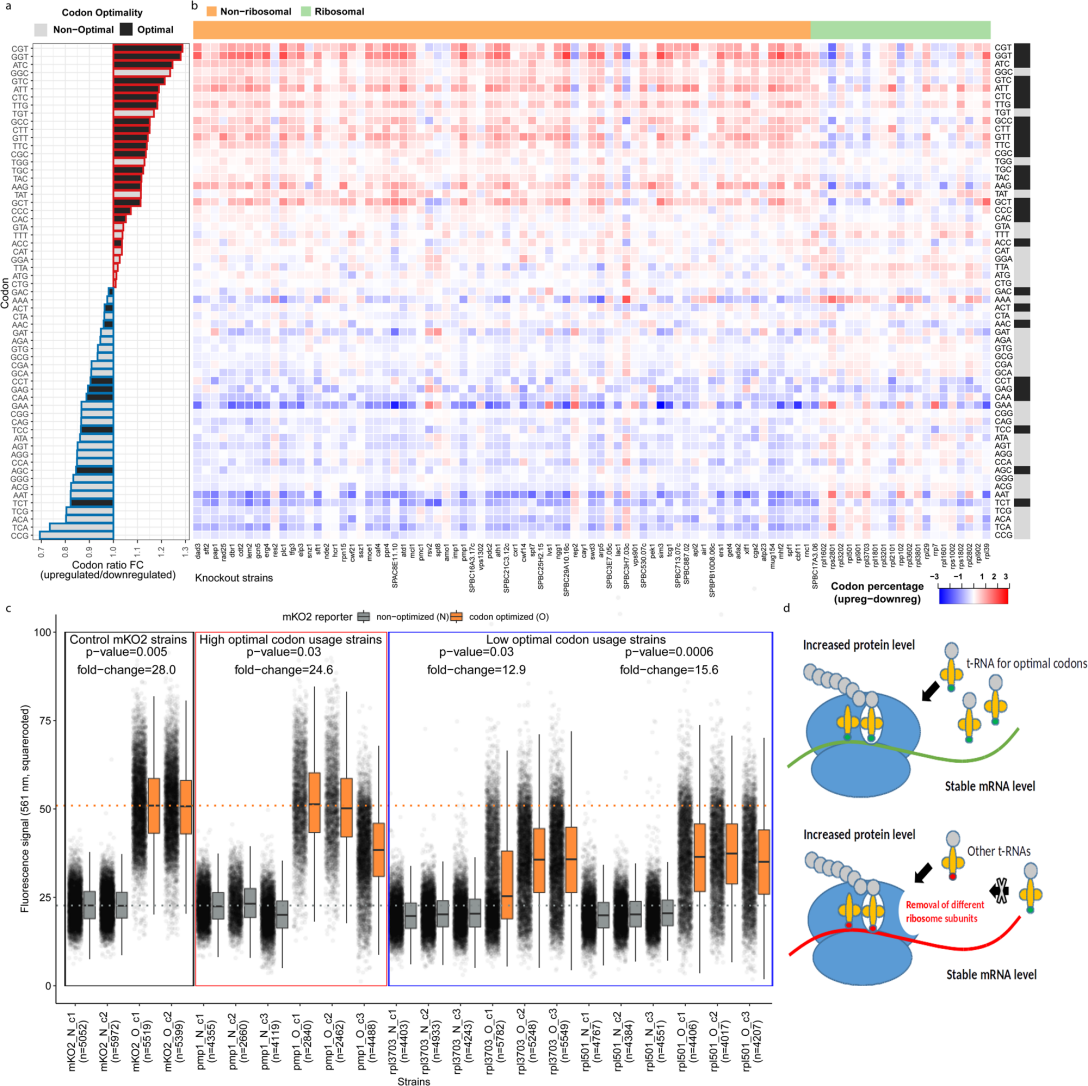
Translationally upregulated genes use optimal codons. **a** Fold-change in codon percentage between the 769 up- and down-regulated proteins with stable mRNA levels. Red boxes depict codons that exhibit an overall trend of proportional increase in upregulated proteins, and blue depicts proportional increase in downregulated proteins. Optimal codons are colored black, while non-optimal codons are colored gray. **b** Heatmap depicting optimal and non-optimal codon usage among the 769 translationally regulated genes (protein change without transcript change) in 93 strains. Red and blue boxes indicate higher codon percentages in upregulated or downregulated proteins, respectively. Optimal codons are marked in black, and non-optimal codons are marked in gray. Ribosome-associated genes are marked in green, and non-ribosomal genes are marked in orange. **c** Boxplot of the fluorescence intensities (square-rooted for normal distribution) measured in strains either containing the codon optimized (O) or the non-optimized (N) mKO2 (Kusabira orange) constructs. *P*-values and fold changes resulted from two-sided Student’s *t*-tests with a CI of 95% between the means of intensities of biologically independent experiments (*n* = 2 for control strains and *n* = 3 for high and low optimal codon strains). Dotted lines indicate the mean values of the control mKO2 strains with N (gray) and O (orange) constructs for easier reference. The interquartile range (IQR) is depicted by the box with the median represented by the centerline. Whiskers maximally extend to 1.5*IQR (with outliers shown). **d** Schematic illustrating that codon usage for translational upregulation of protein levels under stable mRNA conditions might correlate with the structural composition of the ribosome. Source data are provided as a Source Data file.

Furthermore, the paired transcriptome/proteome data allowed us to check the translation efficiency for the 769 genes with stable mRNA levels. Interestingly, we observed clear differences in codon usage between the generally up- and downregulated proteins (Supplementary Fig. 9d, e). Notably, optimal codons^27^ were strongly overrepresented in the translationally upregulated proteins (Fig. 5a). This suggests that protein expression increase under stable mRNA conditions is linked to the more frequent utilization of previously reported high abundance tRNA species in *S. pombe*^27^ for this subset of genes. Supporting this principle, when ribosome-associated genes are deleted, this effect is mitigated (Fig. 5b, Supplementary Fig. 9f). The same trend can be observed when comparing fluorescence protein constructs carrying either optimized or non-optimized codons. Strains that exhibited high optimal codon usage in the previous experiment showed stronger expression of the fluorescent protein, similar to the control strains, while knockout strains that had lower optimal codon usage in our study did not increase expression of the reporter protein that strongly (Fig. 5c). Presumably, for the ribosomal subunit knockout strains, these effects occur due to changes in the translational efficiency of the ribosome itself (Fig. 5d).

In this study, we used quantitative mass spectrometry to measure the proteome of the entire *S. pombe* knockout library containing 3308 knockout strains. Here, we provide a comprehensive proteomics resource for the effect of single gene knockouts in a eukaryotic organism, surpassing any transcriptome screen in yeast to date. We used the protein expression data as a basis for functional annotation and selected 94 knockout strains to study posttranscriptional gene regulation to show that optimal codon usage is one possible scenario for the upregulation of protein expression under stable mRNA conditions. Expression and annotation data, together with mapped homology information for human and *S. cerevisiae*, can be found in an easy-to-access online database.

## Methods

### Yeast culture and cell lysis

For the full screen, the *S. pombe* knockout library (Bioneer, version M-3030H) was grown in YES medium (5 g l^−1^ yeast extract, 30 g l^−1^ D-glucose, supplemented with 225 mg l^−1^ adenine, L-histidine, L-leucine, uracil and L-lysine each f.c.) at 32 °C with shaking at 160 rpm for 2 days. A second inoculation was performed with 5 µl of the initial culture in a 96-deepwell plate filled with 1 ml YES media and grown for another 12–14 h. For the paired transcriptome proteome dataset, 2 ml cultures were grown.

Cells were pelleted by centrifugation for 5 min at 1000 x *g*. The pellet was washed twice with ice-cold Milli-Q water. Lysis was performed by adding 200 µl 8 M urea (#U1250, Sigma Aldrich) in 50 mM (NH_4_)HCO_3_ pH 8.0 (Sigma Aldrich) to the pellet and incubating shaking at 700 rpm for 30 min. Cell debris was removed by centrifugation for 5 min at 1000 x *g*, and the supernatant was transferred into a 96-well plate. The protein concentration was determined with Bio-Rad Protein Assay Dye Reagent (#500-0006, Bio-Rad).

For all strains, the OD600 density values of the initial growth culture (preinoculation) were measured. The Z-score for growth was calculated based on the OD600 density values of each 96-well plate separately by subtracting the mean OD600 value from the OD600 value of each well and dividing by the standard deviation of the OD600 values in the respective 96-well plate.

### TMT labeling

Twenty microliters of the cell lysate, which contained approximately 3.7 µg of protein, was reduced for 30 min with dithiothreitol f.c. 1 mM (#D0632, Sigma Aldrich) and subsequently alkylated for 20 min with iodoacetamide f.c. 5 mM (#I6125, Sigma Aldrich) shaking at 300 rpm at room temperature in the dark. Proteins were diluted in seven volumes of 50 mM (NH_4_)HCO_3_ pH 8.0 and digested overnight with 50 ng endoproteinase Lys-C (#129-02541, Wako) shaking at 300 rpm at 25 °C. Peptides were desalted using StageTips^28^ made from two layers of C_18_ material (#2215, 3 M). Briefly, StageTips were activated using 100% methanol (#20864.320, VWR) and washed with 80% acetonitrile (#20048.320, VWR) in 0.1% formic acid (#5330020050, Millipore). Equilibration and wash steps after peptide loading were performed with 100 mM triethylammonium bicarbonate (TEAB) pH 8.4 (#18597, Sigma Aldrich). Peptides were eluted from StageTips with 80% acetonitrile in 100 mM TEAB, and the organic solvent was subsequently evaporated. The labeling reaction was performed with 8 µg Tandem Mass Tag (#90110, TMT10plex™ Isobaric Label Reagent, Thermo Scientific) for 2 h at room temperature, followed by 15 min quenching with hydroxylamine f.c. 0.4% (#438227, Sigma Aldrich) and acidifying with five volumes 0.1% formic acid. One batch of wt *S. pombe* grown in a 2 ml deep 96-well plate filled with 1 ml YES media was labeled with Tandem Mass Tag 127 C beforehand and frozen in aliquots at −80 °C. An aliquot was combined with every 10plex pool to account for run-to-run and plate-to-plate variability. Each pool of 10 tandem mass tag samples was cleaned by StageTip purification as described above. Peptide elution was performed with 80% acetonitrile in 0.1% formic acid/50 mM (NH_4_)HCO_3_ pH 8.0. After the organic solvent was evaporated, the complete sample was injected for mass spectrometry measurement. In the case of replicates, each replicate was labeled with a different TMT label and measured with different strains to eliminate technical bias.

### Mass spectrometry measurement

Measurements were performed on a Q Exactive Plus mass spectrometer (Thermo Scientific) coupled to a Thermo Scientific EASY-nLC1000 HPLC system (Thermo Scientific). Peptides were separated on self-packed C_18_ columns (#11-1-9.aq.0001, Dr Maisch GmbH) using the following gradient: 0–157 min, 2–22% solvent B; 157–208 min, 22–40% solvent B; 208–212 min, 40–95% solvent B at a flow rate of 225 nl min^−1^. Solvent A consisted of 0.1% formic acid, and solvent B consisted of 80% acetonitrile (#20048.320, VWR) in 0.1% formic acid (#5330020050, Millipore). Peptides were ionized with spray voltages of 2.0–2.4 kV. Data acquisition for TMT samples was performed with Xcalibur 3.1 (Thermo) in positive ion mode with a Top15 data-dependent acquisition method. The full scan was set at a resolution of 70,000 with a scan range of 300–1650 m/z and an AGC target of 3*10^6^. The MS/MS was triggered with a 1.8 m/z isolation window at a minimum intensity threshold of 8.3*10^3^ with the following settings: ion charge 2–7; peptide match preferred; isotope exclusion on and a dynamic exclusion set for 35 s. Fragmentation energy was set to 33, resolution at 35,000 with an AGC target of 1*10^5^ and a maximum injection time of 120 ms.

### mRNA extraction and sequencing

Total RNA extraction was performed according to ref. 29 and the RNA pellet was resuspended in 87 µl of RNase free water. DNA was digested using 3 µl of DNase (#79254, Qiagen) and 10 µl of RDD DNase buffer at 37 °C for 30 min. RNA cleanup was performed with the Purelink RNA Mini kit (#12183018A, Thermo Scientific) according to the manufacturer’s instructions. RNA integrity was measured on a Bioanalyzer 2100 system (#5067-1511, Agilent).

NGS library prep was performed with Lexogen 3´mRNA-Seq Library Prep Kit FWD HT following Lexogen’s standard protocol (015UG110V0110). Libraries were prepared with a starting amount of 338 ng and amplified in 13 PCR cycles. Libraries were profiled using a High Sensitivity DNA chip on a 2100 Bioanalyzer (#5067-4626, Agilent) and quantified using the Qubit dsDNA HS Assay Kit in a Qubit 2.0 Fluorometer (Q32851, Life Technologies). A total of 2 × 192 libraries were pooled together in an equimolar ratio and sequenced in single-end mode on 2 NextSeq 500 high-output flowcells for 1 × 75 cycles plus 2 × 8 cycles for the dual index read.

### MS data analysis

#### Identification and quantitation

All MS raw files were run in a single MaxQuant^30^ version v. 1.6.1.0 using the integrated Andromeda search engine and the Schizosaccharomyces_pombe.ASM294v2.29.pep.all.fa database downloaded from ENSEMBL (www.ensembl.org). Carbamidomethyl on cysteine was set as a fixed modification. Annotation of measured spectra was done using nonparametric simple target-decoy (TD) database searches. Search results of each LC-MS/MS run were filtered at peptide spectrum matches (PSM) FDR < 0.01. Individual protein scores were calculated by summing up Mascot ion scores of the best PSM for all unique peptides of that protein. Proteins were further filtered at protein FDR < 0.01. The second peptide search was disabled, and only unique peptides were considered for quantitation. The mqpar file with all settings is available at ProteomeXchange.

Protein groups with at least one unique peptide were considered for further analysis. Protein groups without intensity values in the technical spike-in sample were removed from the analysis for the respective MS measurement. To ensure sufficient proteome coverage for downstream analysis, samples with less than 1250 identified proteins (one-fourth of the protein-coding genes in *S. pombe*) were repeated.

In the case of replicates, when protein groups were measured in three replicates, the fourth value was imputed based on the Bayesian PCA^31^ missing value estimation method.

#### Labeling performance

Under-labeling was determined by calculating the percentage of evidence without TMT labels after setting TMT as variable modification and removing the contaminants from the analysis^32^. Over-labeling was assessed based on the labeling of H, S, T, and Y amino acids in addition to N-terminal and lysine labeling^32^.

#### Normalization

We used the spiked-in wild-type sample (127 C label) as a global reference to perform initial normalization after logarithmic transformation. Subsequently, we performed sample median normalization, expecting that the average protein intensity should be identical for each sample of the run. To prevent systematic bias of different numbers of peptide identification among MS runs, we assumed that the average intensity of each protein in each run should be identical, and hence, the median intensity of each protein in all the runs was adjusted to the global median intensity of the respective protein. After this normalization step, we did not consider the technical spike-in samples for any further analysis. In our quest to find the most suitable normalization method, we also tried the following methods: “ComBat” (“sva” package); “meancenter”, “standardize”, “ratioa”, “ratiog”, “fabatch”, “combat”, “sva” (“bapred” package); and “limma” (“limma” package).

#### Differential regulation analysis

For the detection of differentially regulated proteins in individual knockout strains, we could not use the standard *t*-test as we did not have replicate data. Instead, we used the 461 biological wild-type replicates across all MS runs to calculate the reference mean and standard deviation for each protein. Using a two-sided *z*-test and these reference distributions we calculated the z-score of each individual protein intensity for all knockout measurements. These z-scores were then transformed into *p*-values using the *z*-table. *P*-values were corrected for multiple testing using the Benjamini–Hochberg approach resulting in FDR values. Proteins with FDR < 0.05 were assumed to be differentially regulated in the respective knockout. For all protein datasets with replicates, we used the standard two-sided Student’s *t*-test to test for differential regulation. The differential regulation threshold was set to log2-fold change less than −0.2 or larger than 0.2 with *p*-values less than 0.03 for all measured samples.

### Transcriptome data analysis

Demultiplexed reads were assigned to corresponding samples, for which a minimum of 1.5 million reads per sample were measured. The raw sequencing files were analyzed further using the “NGSpipe2go” RNA-seq pipeline (https://github.com/imbforge/NGSpipe2go). Version of the tools used were R (3.5.1), FastQC (0.11.8), STAR (2.6.1b), SAMtools (1.5), HTSeq (0.9.0), Subread (1.6.2), BEDTools (2.27.1), Picard (2.17.6), RSeQC (3.0.0), Qualimap (2.2.1), KentUtils (v365), rMATS (4.0.2), FastQScreen (0.12.2), deepTools (3.1.0), BamUtil (1.0.13), and STAR-Fusion (0.8.0). The default RPKM calculation was replaced with CPM (counts per million) as 3’-end sequencing was performed. “ASM294v2.40.gtf” was used as the gene model, and “ASM294v2.dna.toplevel.fa” was used as the genome for *S. pombe*. The “rld” transformation of the data from the “DE_Seq” part of the pipeline was replaced with the “vst” transformation due to the high number of samples being processed.

Afterward, the CPM values below the lowest quantile of all CPM values (CPM < 1.006722) were removed before further analysis. The CPM values were log-transformed, and in the case of three measured values, the fourth value was imputed using the Bayesian PCA method as done for the replicate proteome measurements. The differential regulation threshold was set to a minimum log2 fold change of 0.6 and a maximum p-value of 0.005.

### Protein co-expression and clustering

Pearson’s correlation coefficient for each pair of proteins was calculated using the z-score transformed intensity values, while only the complete pairwise observations were considered for calculations.

Clustering was performed using the Kohonen package in R, with the “supersom” function^23^ using the z-score values. We initially grouped the proteins into 144 supersom clusters (maximizing connectivity score), and based on the similarities within and between the clusters and the size of the clusters, we filtered these 144 clusters into 113 clusters.

STRING database version v11 was used for the analysis. Pairs of proteins with a combined score higher than 150 were considered to be associated. The sample size of the random sampling was determined based on the size distribution of the clusters.

### Strain similarity measures and clustering

Pearson’s correlation coefficients for each pair of strains were calculated using the z-score transformed intensity values, while only the complete pairwise observations were considered for calculations.

Clustering was performed using the Kohonen package in R, with the “supersom” function^23^ using the z-score values. We initially grouped the strains into 121 supersom clusters. Then, we removed the 40 clusters containing any wild-type measurements. Afterward, based on the similarities within and between the clusters, we filtered these remaining 81 clusters into 48 clusters with low mean distances to the center of the respective cluster (<75 percentile of all distances) containing 2177 of the 3308 knockout strains.

The STRING database (version v11), Reactome pathways and Reactome reactions (downloaded on June 3rd 2020) were used for the analysis. Pairs of proteins with a combined score higher than 150 were considered to be associated. The sample size of the random sampling was determined based on the size distribution of the clusters.

### Assessment of genes regulated at the protein level

Genes regulated at the protein level only were selected among the genes with measured transcripts and proteins in the respective strains, and only those regulated at the protein level without any significant change at the mRNA level were considered for clustering. Here, only the top 50% of genes (769) were used for clustering, referring to those showing changes only at the protein level of a higher number of knockout strains. The “pamk” function from the “fpc” package of R was used for clustering and defining the optimal number of clusters, which was two.

### Codon optimality analysis

For each knockout strain, we used the open reading frames of the group of up- and downregulated genes with no changes at the RNA level and their respective protein levels to calculate the usage percentage for each of the 64 codons. These percentages were used throughout the analysis to assess significant differences in codon usage between up- and downregulated genes. Codon optimality was assigned based on previously published results^27^.

### Codon optimized orange fluorescent experiments

*S. pombe* strains containing a HYGR marked cassette with ade6p driving mKO2 that is either *S. pombe* codon optimized using commercial algorithms^33^ (LifeTech/Thermo Fisher) or nonoptimized were crossed with deletion mutants (G418R marked) and progeny selected after mating and germination by random spore selection. Briefly, cells were mated on maltose extract (ME) nitrogen-starved plates for 3 days at 25 °C and germinated on rich YS plates overnight. Cells were vortexed in water for 30 s and then plated at low density on YS + HYG + G418 plates for 2 days at 32 °C to select for mating products^34^. Three independent HYGR/G418R double-positive colonies were selected by random spore selection. For flow cytometry experiments, parent strain clones (*N* = PAS916, O = PAS917) and target strains were grown in YS overnight in 96 well plates at 32 °C, diluted into YS 1:50 the next day, and grown another 6 h into log phase^34^. Cells were size-gated for young G2 cells (“head of the comet” in a linear forward versus side scatter plot^34^) to avoid artifacts arising from heterogeneous cell sizes. The orange fluorescence in these populations (~2500–6000 cells) was measured for the three replicates per mutant strain and two replicates for wild-type mKO2 optimized and nonoptimized controls.

Control strains included: PAS916 (non-codon optimized - N) [ade6p:mKO2:ura4t hygMX at Locus2 (between SPBC1711.11 and SPBC1711.12); ura4-D18; leu1-32; ade6-M216; his7-366; h(-)], PAS917 (codon optimized - O) [ade6p: pombe codon optimized mKO2:ura4t hygMX at Locus2 (between SPBC1711.11 and SPBC1711.12); ura4-D18; leu1-32; ade6-M216; his7-366; h(-)].

### Genetic interaction data

Genetic interaction data were obtained from a previous study^16^. Pearson’s correlation coefficient (R) was calculated using “pairwise-complete-observations”. The difference in distribution of Pearson’s correlation coefficient between pairs of proteins belonging to the same or different SOM clusters was tested using the Wilcoxon test.

### Correlation analysis of paired transcriptome and proteome data

Three different measures were assessed using paired transcriptome and proteome data correlations. To accomplish this, the following steps were performed: log2-fold change of individual genes was calculated with respect to the wild-type strain (for transcriptome and proteome), and these log2-fold change values were used for calculation of the correlation coefficient. The first measure was the assessment of pairwise similarity between knockout strains to investigate the effect of different types of data being used (proteome or transcriptome). For this purpose, the pairwise correlation between the strains was calculated using either the transcriptome or proteome data. The second measure was the assessment of the individual knockout strain based on the changes in the transcriptome and proteome in the absence of the respective gene. For this purpose, the correlation for each strain was calculated using the transcriptome to the proteome data. The third measure was the assessment of the individual genes that were measured based on the changes in the transcriptome and proteome in the absence of these 94 knockout strains. For this purpose, the correlation for each measured gene was calculated using the transcriptome or proteome data in these 94 knockout strains. Pearson’s correlation coefficient (R) was calculated using “pairwise-complete-observations” in all three cases.

### Reporting summary

Further information on research design is available in the Nature Research Reporting Summary linked to this article.

## Supplementary information

Supplementary Information

Reporting Summary

## Data Availability

The proteomics data generated in this study have been deposited in the proteomeXchange database under accession code PXD024332 (genome-wide screen) and PXD024383 (94 gene set). The transcriptomics data generated in this study have been deposited in the GEO database under accession code GSE167543 (94 gene set). Source data are provided with this paper.

## Source data

Source Data

## Code availability

Code is available in github (https://github.com/m89ozturk/SpombeDeletionLibraryPaper).

## References

[1] Buccitelli C, Selbach M (2020). mRNAs, proteins and the emerging principles of gene expression control. Nat. Rev. Genet.

[2] Kemmeren P (2014). Large-scale genetic perturbations reveal regulatory networks and an abundance of gene-specific repressors. Cell.

[3] Frejno M (2020). Proteome activity landscapes of tumor cell lines determine drug responses. Nat. Commun..

[4] Müller JB (2020). The proteome landscape of the kingdoms of life. Nature.

[5] Nusinow DP (2020). Quantitative proteomics of the cancer cell line encyclopedia. Cell.

[6] Stefely JA (2016). Mitochondrial protein functions elucidated by multi-omic mass spectrometry profiling. Nat. Biotechnol..

[7] Giaever G (2002). Functional profiling of the Saccharomyces cerevisiae genome. Nature.

[8] Yanagida M (2002). The model unicellular eukaryote, Schizosaccharomyces pombe. Genome Biol..

[9] Rhind N (2011). Comparative functional genomics of the fission yeasts. Science.

[10] Lock A (2019). PomBase 2018: user-driven reimplementation of the fission yeast database provides rapid and intuitive access to diverse, interconnected information. Nucleic Acids Res..

[11] Shetty A (2017). Spt5 plays vital roles in the control of sense and antisense transcription elongation. Mol. Cell.

[12] Finet O (2022). Transcription-wide mapping of dihydrouridine reveals that mRNA dihydrouridylation is required for meiotic chromosome segregation. Mol. Cell.

[13] Booth GT, Wang IX, Cheung VG, Lis JT (2016). Divergence of a conserved elongation factor and transcription regulation in budding and fission yeast. Genome Res.

[14] Schmidt MW, Houseman A, Ivanov AR, Wolf DA (2007). Comparative proteomic and transcriptomic profiling of the fission yeast Schizosaccharomyces pombe. Mol. Syst. Biol..

[15] Carpy A (2014). Absolute proteome and phosphoproteome dynamics during the cell cycle of Schizosaccharomyces pombe (Fission Yeast). Mol. Cell Proteom..

[16] Ryan CJ (2012). Hierarchical modularity and the evolution of genetic interactomes across species. Mol. Cell.

[17] Frost A (2012). Functional repurposing revealed by comparing S. pombe and S. cerevisiae genetic interactions. Cell.

[18] Kim D-U (2010). Analysis of a genome-wide set of gene deletions in the fission yeast Schizosaccharomyces pombe. Nat. Biotechnol..

[19] Thompson A (2003). Tandem mass tags: a novel quantification strategy for comparative analysis of complex protein mixtures by MS/MS. Anal. Chem..

[20] Brenes A, Hukelmann J, Bensaddek D, Lamond AI (2019). Multibatch TMT reveals false positives, batch effects and missing values. Mol. Cell Proteom..

[21] Kustatscher G (2019). Co-regulation map of the human proteome enables identification of protein functions. Nat. Biotechnol..

[22] Szklarczyk D (2019). STRING v11: protein-protein association networks with increased coverage, supporting functional discovery in genome-wide experimental datasets. Nucleic Acids Res..

[23] Wehrens R, Kruisselbrink J (2018). Flexible self-organizing maps in kohonen 3.0. J. Stat. Softw..

[24] Vo TV (2016). A proteome-wide fission yeast interactome reveals network evolution principles from yeasts to human. Cell.

[25] Jassal B (2020). The reactome pathway knowledgebase. Nucleic Acids Res.

[26] Payne SH (2015). The utility of protein and mRNA correlation. Trends Biochem Sci..

[27] Harigaya Y, Parker R (2016). Analysis of the association between codon optimality and mRNA stability in Schizosaccharomyces pombe. BMC Genomics.

[28] Rappsilber J, Mann M, Ishihama Y (2007). Protocol for micro-purification, enrichment, pre-fractionation and storage of peptides for proteomics using StageTips. Nat. Protoc..

[29] Schmitt ME, Brown TA, Trumpower BL (1990). A rapid and simple method for preparation of RNA from Saccharomyces cerevisiae. Nucleic Acids Res.

[30] Cox J, Mann M (2008). MaxQuant enables high peptide identification rates, individualized p.p.b.-range mass accuracies and proteome-wide protein quantification. Nat. Biotechnol..

[31] Stacklies W, Redestig H, Scholz M, Walther D, Selbig J (2007). pcaMethods–a bioconductor package providing PCA methods for incomplete data. Bioinformatics.

[32] Zecha J (2019). TMT labeling for the masses: a robust and cost-efficient, in-solution labeling approach. Mol. Cell Proteom..

[33] Al-Sady B, Greenstein RA, El-Samad HJ, Braun S, Madhani HD (2016). Sensitive and quantitative three-color protein imaging in fission yeast using spectrally diverse, recoded fluorescent proteins with experimentally-characterized in vivo maturation kinetics. PLoS One.

[34] Greenstein, R. A. et al. Noncoding RNA-nucleated heterochromatin spreading is intrinsically labile and requires accessory elements for epigenetic stability. *Elife* **7**; 10.7554/eLife.32948 (2018). 10.7554/eLife.32948 PMC6070336 30020075

