# Supplementary material for: Proteome effects of genome-wide single gene perturbations
Source: Nat Commun. 2022 Oct 18;13:6153. doi: 10.1038/s41467-022-33814-8 (PMC9579165; doi:10.1038/s41467-022-33814-8)
Supplement: Supplementary file 1 — Supplementary Information [file 41467_2022_33814_MOESM1_ESM.pdf]

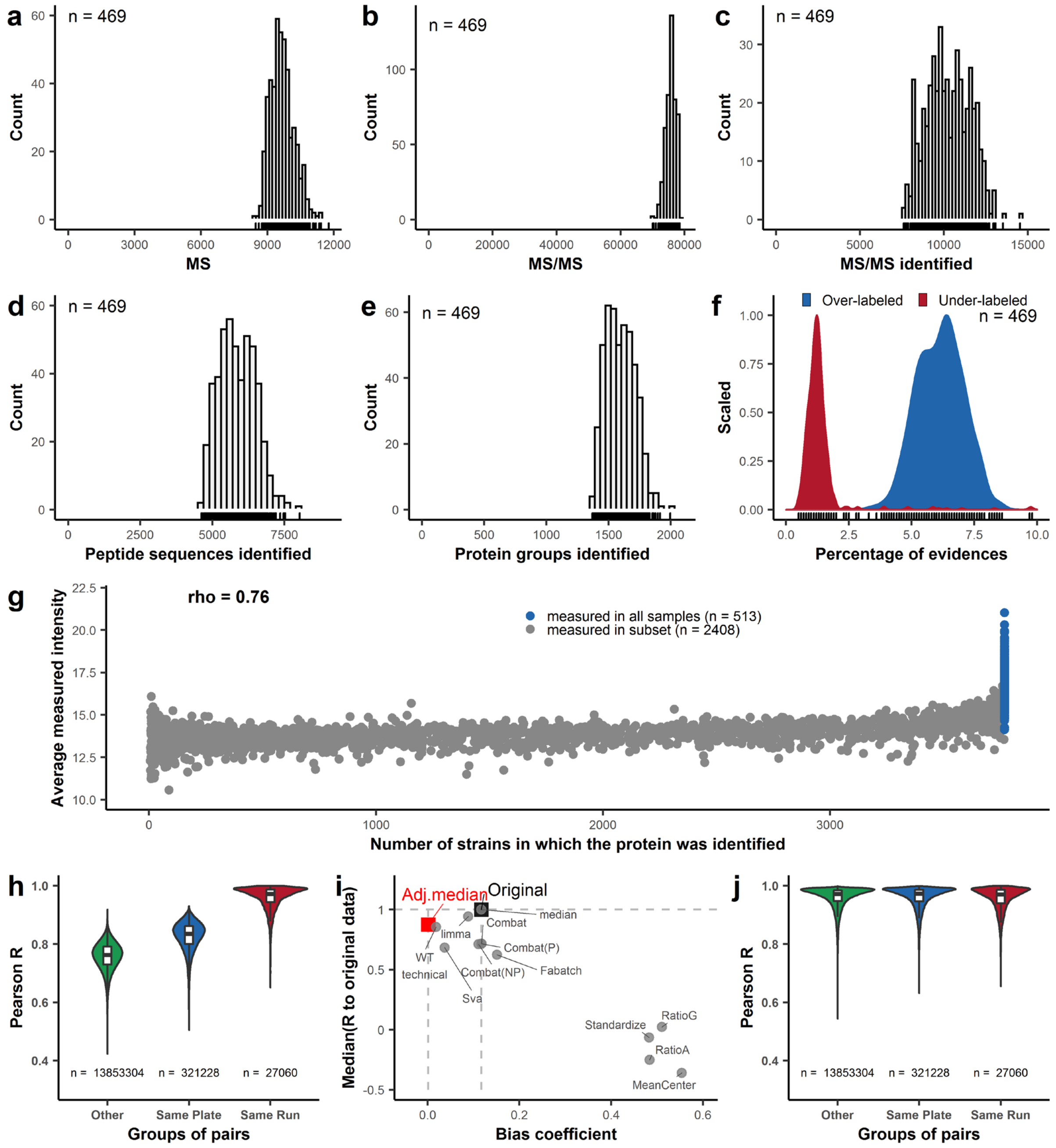

Supplementary Figure 1: Quality control and normalization of the proteomics data. a-e, Histograms showing high similarity of the 469 mass spectrometry runs by number of acquired MS spectra; number of triggered MS/MS spectra; number of identified MS/MS spectra and the number of identified peptides and protein groups. **f,** Fractions of TMT under-labeled or putative over-labeled peptide evidence for *S. pombe* proteins. **g,** Scatter plot of the number of times a protein group was identified among all samples and the mean of its intensity. Marked in blue are the 513 protein groups, which were measured in all samples and used for assessing data normalization. **h,** Distribution of correlation coefficients between proteomes before normalization, emphasizing the strong tendency of samples from the same run to be most highly correlated. The number of pairs included in each group of pairs is depicted in the plot as n. **i,** Dimensional plot correlating the bias coefficient (the slope of the line fitted to the median of the correlation coefficients) with the similarity to the original data after different normalization methods. Original data (black square) and normalized data after applying the adjusted median normalization method (red square) are highlighted. **j,** The distribution of correlation coefficients between different proteomes after applying adjusted median normalization (see methods) emphasizing strongly minimized observed batch effects. The number of pairs included in each group of pairs are depicted in the plot as n. **h and j,** The interquartile range (IQR) is depicted by the box with the median represented by the center line. Whiskers maximally extend to 1.5*IQR.

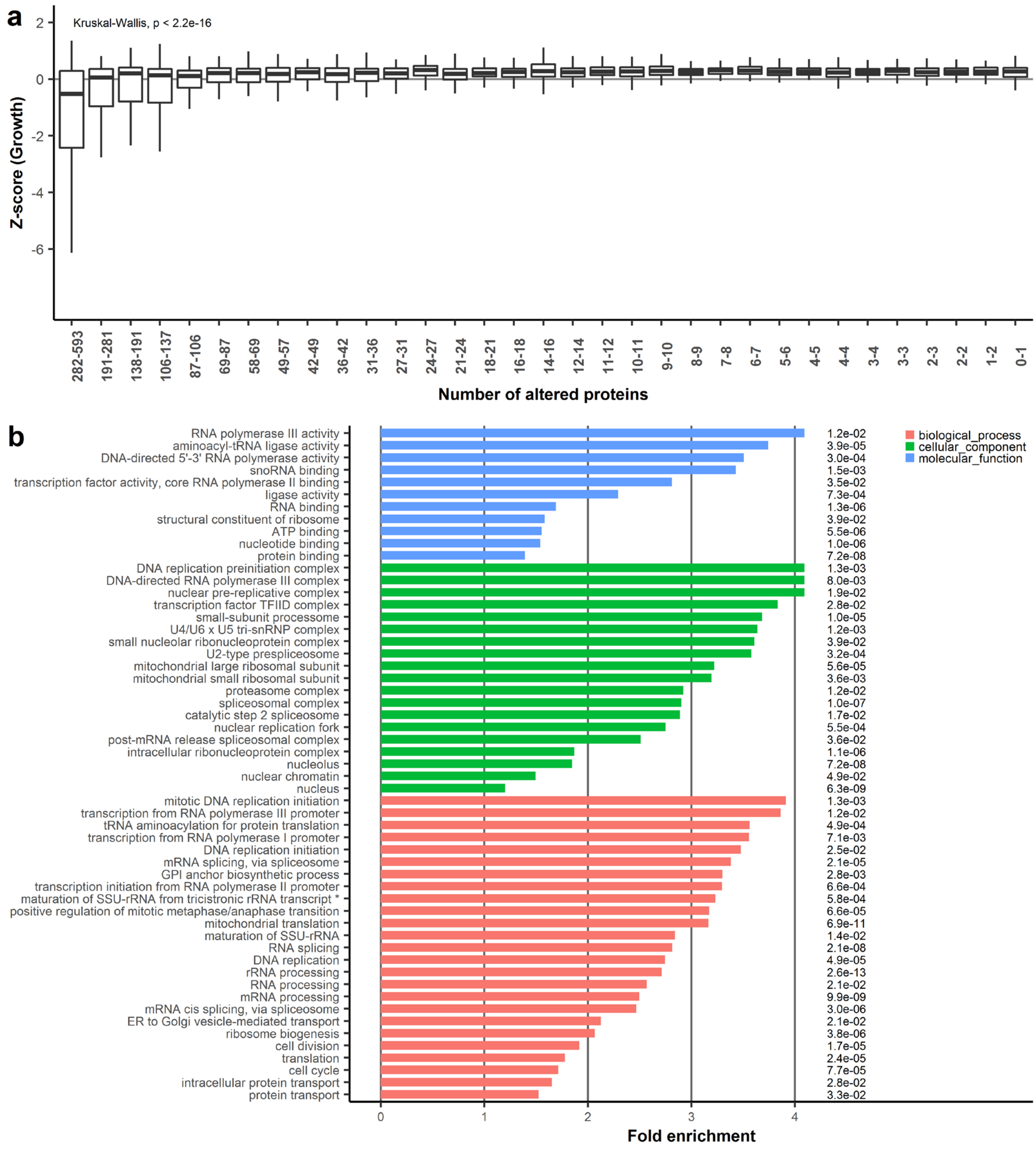

Supplementary Figure 2: Growth phenotypes and essential genes. a, Growth behavior of the knockout strains grouped when binned into 33 groups by the number of altered proteins (each bin contains 100 strains). The interquartile range (IQR) is depicted by the box with the median represented by the center line. Whiskers maximally extend to 1.5*IQR. **b,** Functional enrichment analysis of essential genes in *S. pombe*, where permutation based p-values are shown to the right of each bar

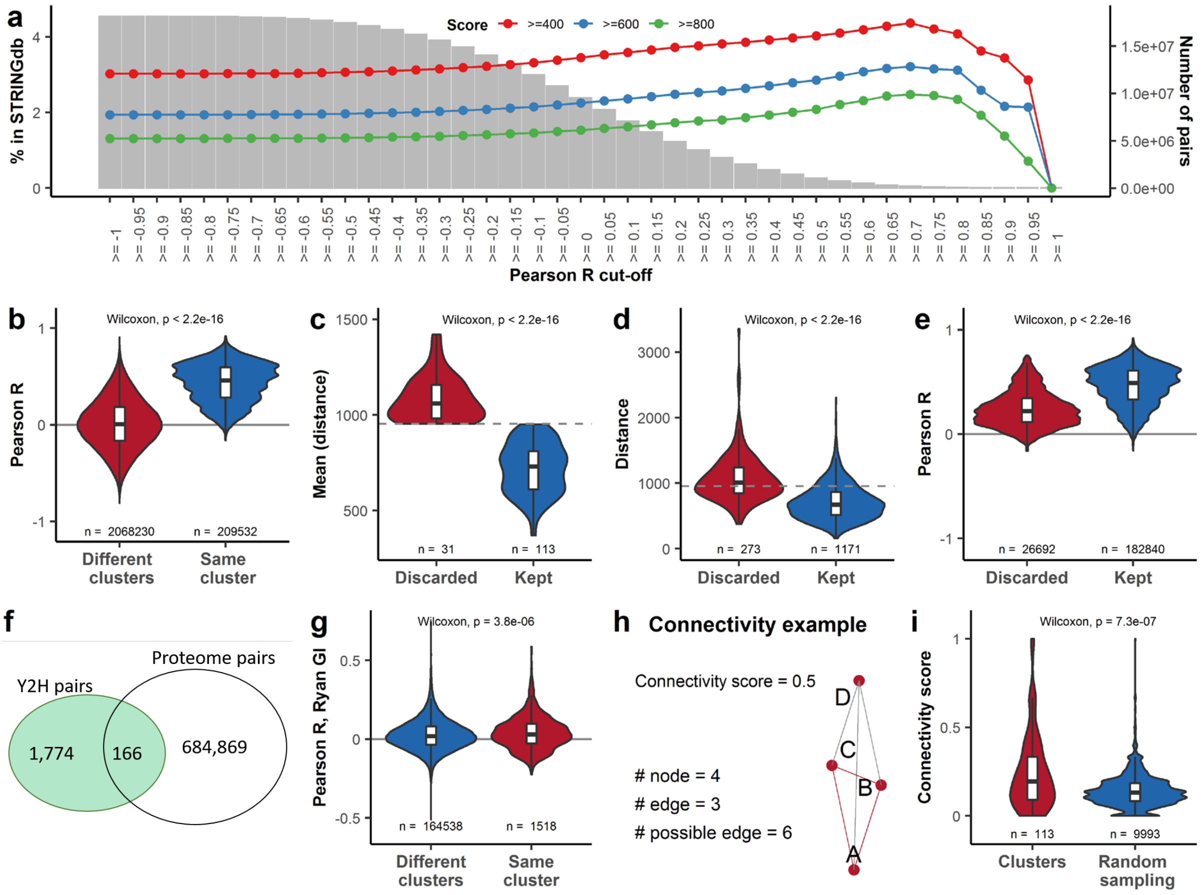

Supplementary Figure 3: Validation of the supersom clustering for protein co-expression. a, Fraction of protein pairs that correspond to protein-protein interactions in the STRING database by increasing protein co-expression. Pearson correlation coefficient (R) cut-offs are depicted as colored dots. Score thresholds (color of the dots) reflect the STRINGdb combined score. Gray bars represent the number of pairs included in the analysis per Pearson R cut-off (y-axis on the right side). **b**, Distributions of the Pearson R between individual protein pairs based on whether they belong to the same or to different SOM clusters. Overall, proteins were clustered efficiently, resulting in higher correlation coefficients between proteins in the same cluster in comparison to proteins from different clusters. **c**, Distributions of the mean distance to the center of the respective cluster generated by the supersom algorithm. Clusters with a large mean distance (>75 percentile, dashed line) were discarded, resulting in 113 high-confidence clusters. The number of clusters included in each category is depicted in the plot as n. **d**, Difference in the distribution of each individual protein's distance to its cluster center based on whether the cluster was kept or discarded in (**c**). The number of proteins included in each category is depicted in the plot as n. **e**, Distributions of Pearson correlation coefficients for protein pairs from the same supersom cluster according to whether the clusters were kept or discarded. **f**, Venn diagram illustrating the overlap between possible interactions from the retained clusters of the proteome dataset and reported yeast two-hybrid interactions. **g,** Distributions of Pearson correlation coefficients of published genetic interaction data for protein pairs according to whether the pairs belong to the same supersom cluster or not. **h**, Illustration explaining the connectivity score: the four nodes depict proteins, and the three interactions are represented by red edges leading to a connectivity score of 0.5 (3 out of 6 possible interactions). **i**, Distributions of the connectivity scores (percentage of all potential interactions previously annotated for each cluster, scaled from 0-1) based on interactions defined in the STRING database. The 113 high-confidence clusters' "connectivity score" is twice as high as expected when compared to random sampling. The number of clusters included in each category is depicted in the plot as n. **b-e, g and i,** The interquartile range (IQR) is depicted by the box with the median represented by the center line. Whiskers maximally extend to 1.5*IQR. Statistical significance was determined by using two-sided unpaired-samples Wilcoxon rank sum test with a CI of 95%. **b, e and g**, The number of pairs included in each group of pairs is depicted in the plot as n.

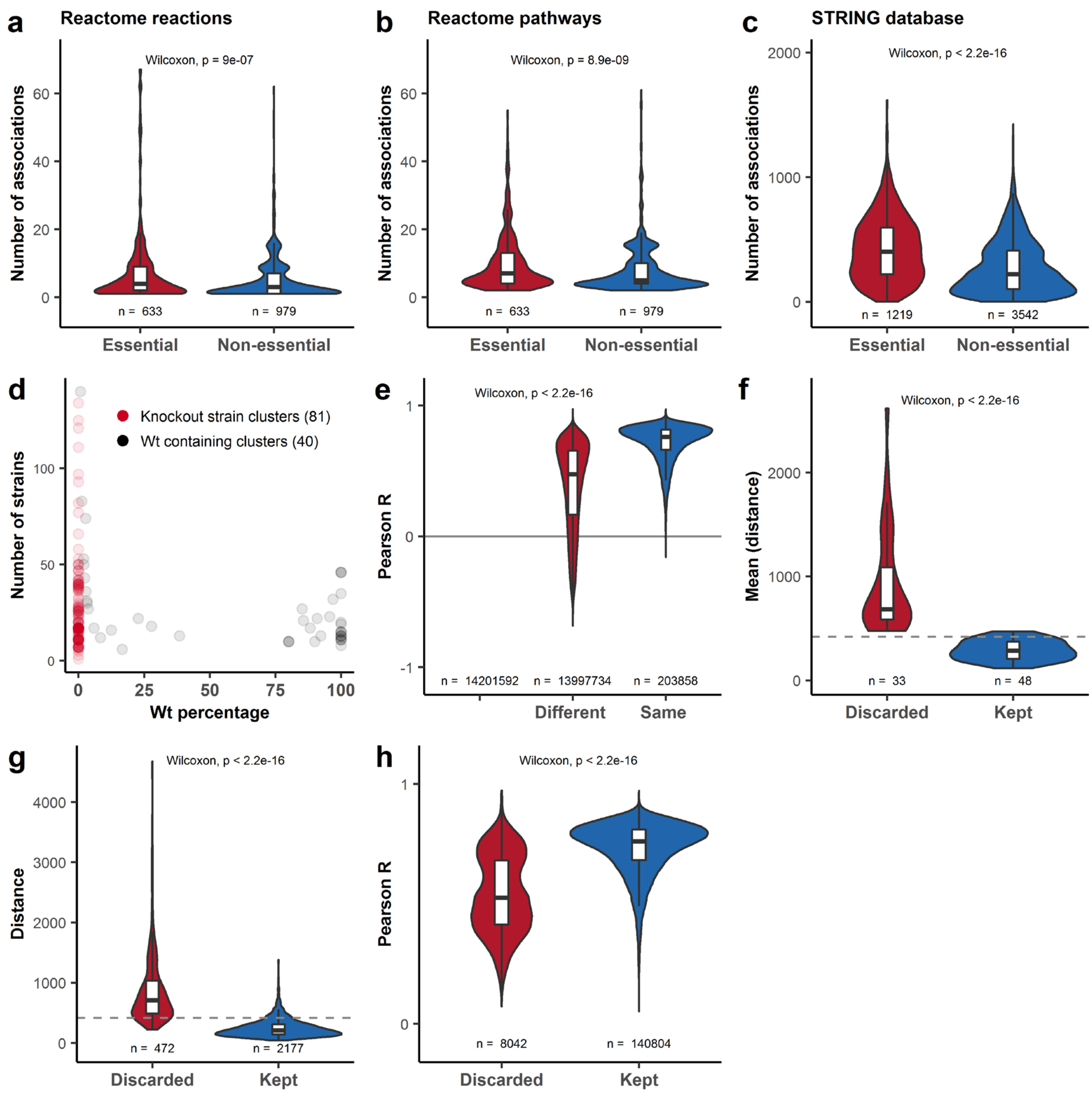

Supplementary Figure 4: Validation of the supersom clustering for strain similarity. a-c, Number of associations per gene for Reactome reactions, Reactome pathways and STRING database associations for essential and nonessential genes. The number of genes in each category is depicted in the plot by n. **d,** Scatterplot showing the percentage of wild-type strains in the initial 121 supersom clusters. Only clusters without wild-type strains (marked red) were kept for further analysis. **e,** Distributions of pairwise Pearson correlation coefficients for the pairs of strains within the same or across different supersom clusters. The number of pairs included in each group of pairs is depicted in the plot as n. **f**, Distributions of the mean distances to the center of each cluster comparing discarded and kept clusters. Clusters with a large mean difference (>75 percentile, dashed line) were discarded, resulting in 48 high-confidence clusters. The number of clusters included in each category is depicted in the plot as n. **g**, Violin and box plot showing the distribution of the distance of individual strains to their cluster center comparing discarded and kept clusters. The number of strains included in each category is depicted in the plot as n. **h**, Distributions of Pearson correlation coefficients for pairs of strains from discarded and kept supersom clusters. The number of pairs included in each group of pairs is depicted in the plot as n. **a-c and e-h,** The interquartile range (IQR) is depicted by the box with the median represented by the center line. Whiskers maximally extend to 1.5*IQR. Statistical significance was determined by using two-sided unpaired-samples Wilcoxon rank sum test with a CI of 95%.

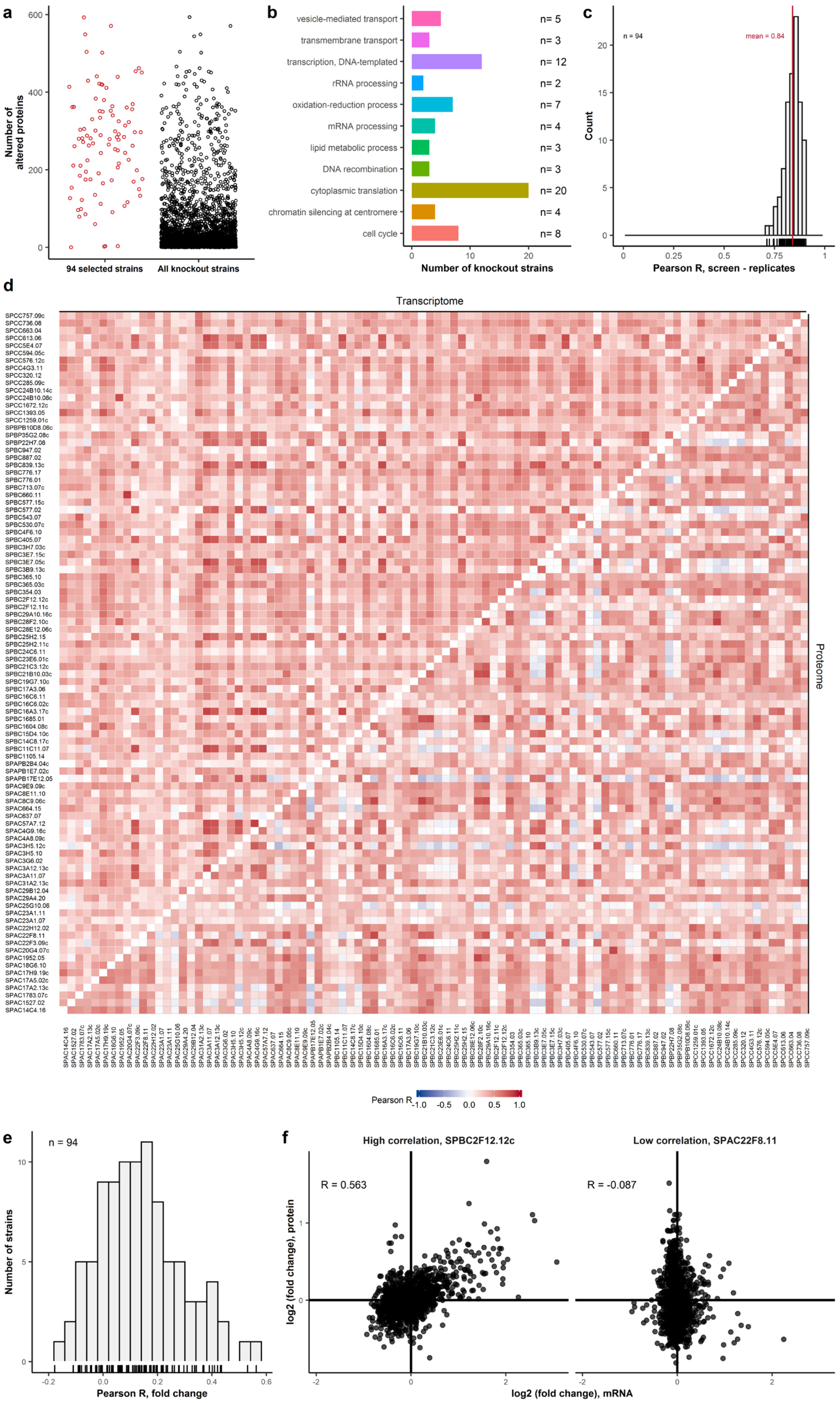

Supplementary Figure 5: Analyzing the replicate proteomes of 94 selected knockout strains. **a**, Dotplots showing the number of altered proteins from the library-wide screen of the 94 selected knockout strains and of all knockout strains. **b**, Gene ontology annotations of the selected knockout strains for paired transcriptome and proteome measurement. **c**, Histogram depicting the distribution of Pearson correlation coefficients for comparison between initial quantitation in the library screen and the re-measurement in quadruplicates for each of the 94 strains. **d**, Heatmap of Pearson correlation coefficients between pairs of strains at the proteome level (bottom-right) and at the transcriptome level (top-left). **e**, Histogram showing the distribution of the Pearson correlation coefficients between mRNA and protein expression for each individual knockout strain. **f**, Scatter plots for two examples, one knockout showing high (left panel) and the other low (right panel) correlation of transcript-protein abundance changes when compared to wild-type. Blue lines show linear fitting of the data with the 95% confidence interval in grey shade.

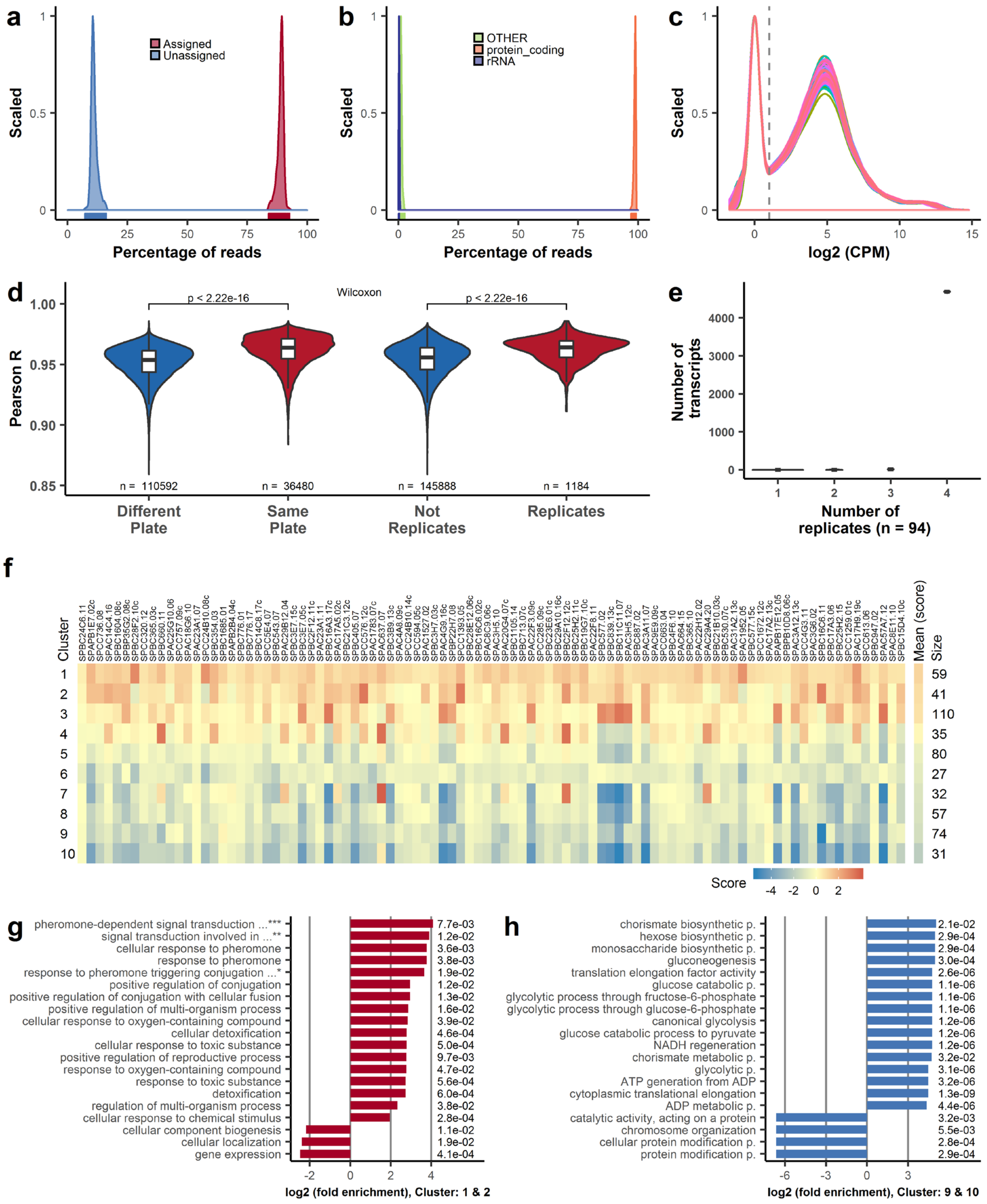

Supplementary Figure 6: Analyzing the replicate transcriptomes of 94 selected knockout strains. a, Scaled density plots showing the distributions of the uniquely assigned (red) and unassigned (blue) read in percentages. **b,** Distribution of the reads per gene type (protein-coding in orange, ribosomal RNA in dark blue and all others combined in light green). **c**, Scaled density plot showing the distributions of the log2-transformed counts per million (CPM) of the transcripts in each sample, n=384; the vertical dashed line indicates the filtering threshold. **d**, Distributions of Pearson correlation coefficients for pairs of strains grown in the same plate or biological replicates of a knockout strain. The interquartile range (IQR) is depicted by the box with the median represented by the center line. Whiskers maximally extend to 1.5*IQR. Statistical significance was determined by using two-sided unpaired-samples Wilcoxon rank sum test with a CI of 95%. The number of pairs included in each group of pairs is depicted in the plot as n. **e**, Distribution of the number of identified transcripts across the replicates. **f**, Heatmap of k-means clustered genes differentially regulated in at least 6 of the 94 strains to characterize changes at the transcript level. Score represents the mean differential regulation score of the genes of each group (score is -log10(p-value) multiplied by the sign of the fold change), ordered by average score. **g and h**, The top 20 biological process and molecular function gene ontology terms (by absolute log2 fold change) enriched in (**i**), mostly upregulated transcripts (clusters 1 and 2) and (**h**), mostly downregulated transcripts (clusters 9 and 10). Gene set enrichment p-values and fold enrichments were calculated using Fisher's exact test.

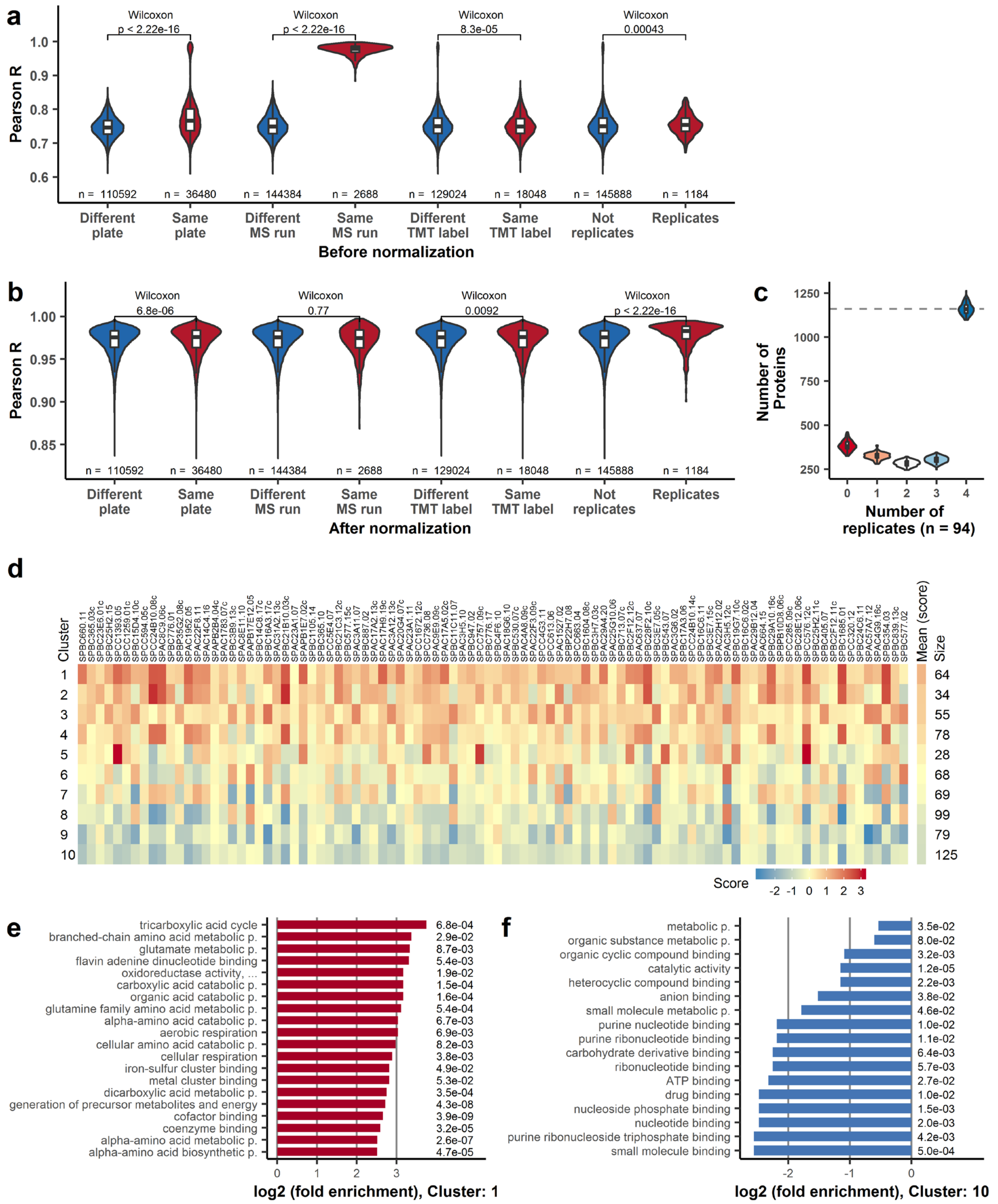

Supplementary Figure 7: Analyzing the proteome of 94 selected knockout strains. a-b, Distributions of Pearson's correlation coefficients calculated from protein expression values between the strains (**a**) before and (**b**) after using adjusted median normalization. Statistical significance was determined by using two-sided unpaired-samples Wilcoxon rank sum test with a CI of 95%. **c**, Distribution of the number of identified proteins across the replicates. **d**, Heatmap of sorted k-means protein clusters from proteins measured in at least 64 of the 94 strains to characterize changes at the protein level when differentially regulated in at least 9 strains. Score represents the mean differential regulation score of the proteins of each group (score is -log10(p-value) multiplied by the sign of the fold change), ordered by average score. **e and f**, The top 20 biological process and molecular function gene ontology terms (by absolute log2 fold change) enriched in (**e**), mostly upregulated proteins (cluster 1) and (**f**), mostly downregulated proteins (cluster 10). Gene set enrichment p-values and fold enrichments were calculated using Fisher's exact test. **a-c,** The interquartile range (IQR) is depicted by the box with the median represented by the center line. Whiskers maximally extend to 1.5*IQR.

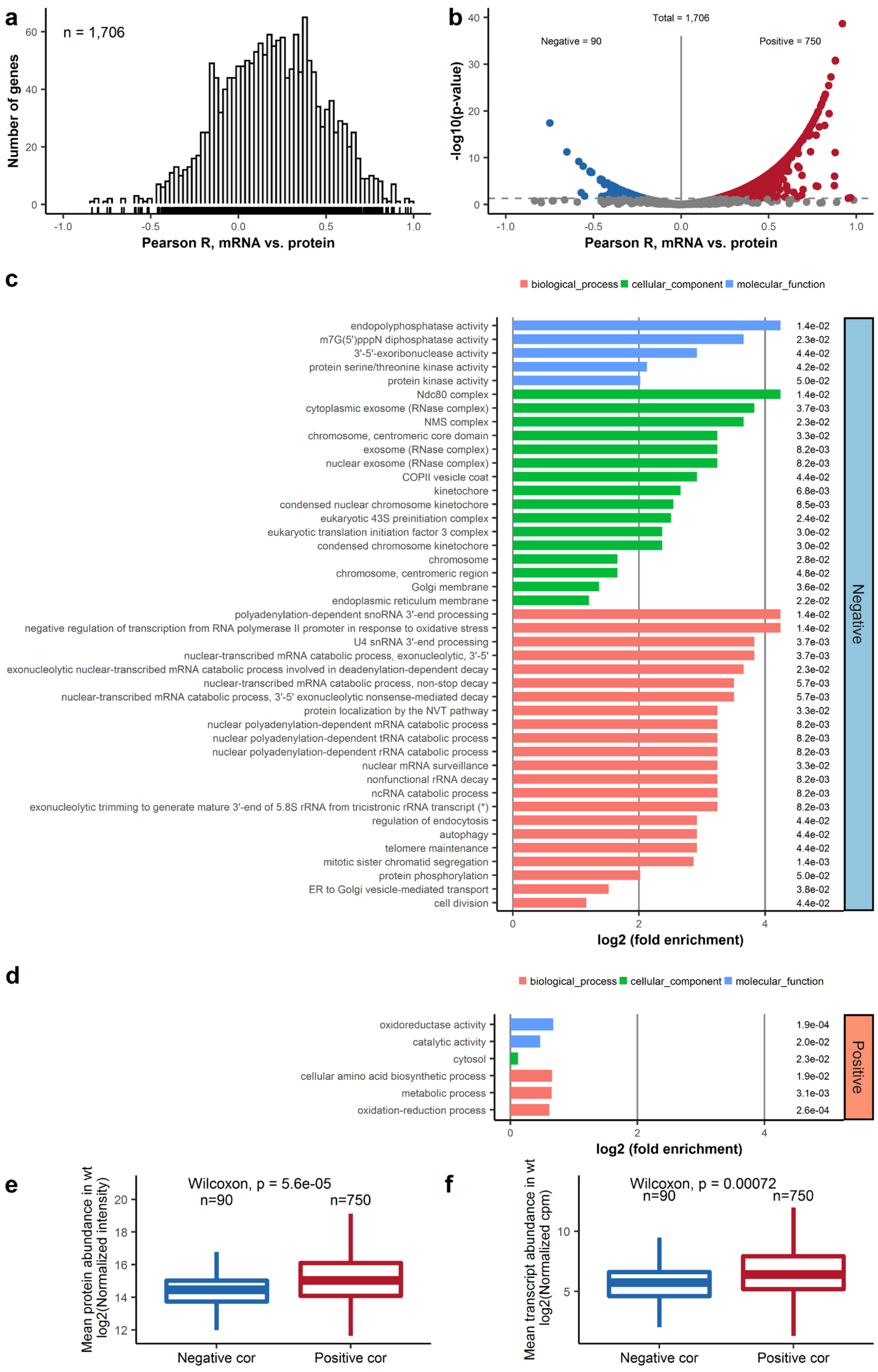

Supplementary Figure 8: Assessing post-transcriptional gene regulation by transcriptome/proteome correlation. a, Histogram showing the distribution of the Pearson correlation coefficients between fold changes of transcript and protein for individual genes (n=1,706). **b**, Volcano plot showing the Pearson correlation coefficients between fold changes of transcript and protein of individual genes (n=1,706) including the respective p-values. Red and blue dots depict statistically significant correlations (p<0.05). **c and d**, Enriched gene ontology terms in the subgroup of genes exhibiting statistically significant negative (**c**) and positive (**d**) Pearson correlations for transcript and protein expression levels. Gene set enrichment p-values and fold enrichments were calculated using Fisher's exact test. **e and f**, Boxplots depict the distribution of the mean protein (**e**) and transcript (**f**) abundance of the negatively and positively correlated set of genes. The interquartile range (IQR) is depicted by the box with the median represented by the center line. Whiskers maximally extend to 1.5*IQR. Statistical significance was determined by using two-sided unpaired-samples Wilcoxon rank sum test with a CI of 95%. The number of genes included in each category is depicted in the plot as n.

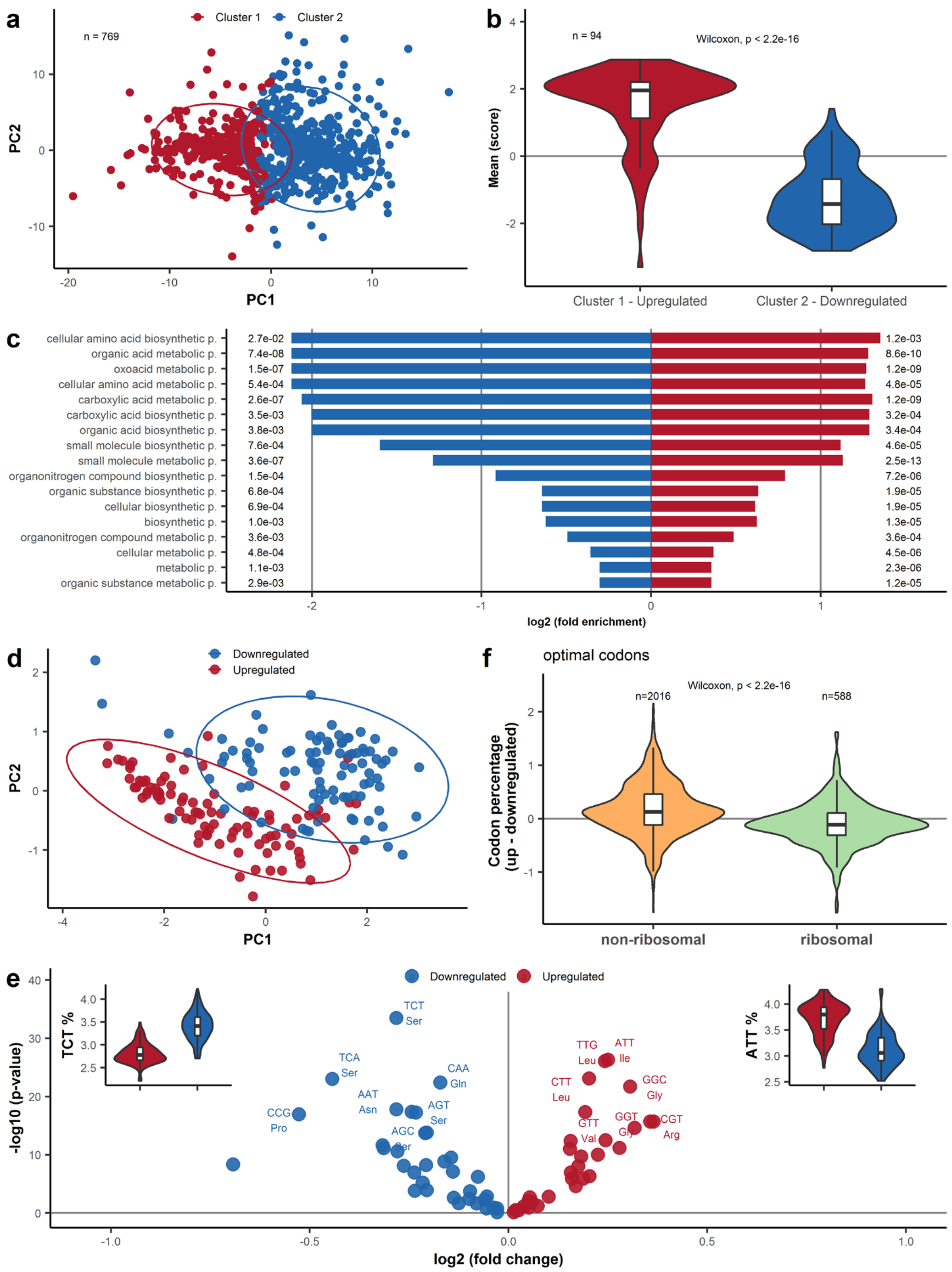

Supplementary Figure 9: Assessing post-transcriptional gene regulation by focusing on genes regulated at the protein level identifies unique codon usage signals. a, Principal component plot showing two clusters of genes based on their regulation similarity at the protein level (genes without mRNA abundance changes) as determined by PAM clustering. The 769 individual dots are colored red or blue based on whether they belong to cluster 1 or 2, respectively. These sets are also emphasized with concentration ellipses depicting normal probability. **b**, Mean differential regulation score (-log10(p-value of differential regulation) multiplied by the sign of the fold change) for the clusters of genes shown in (**a**) for each of the 94 strains. **c**, Common biological process gene ontology terms significantly enriched in generally up- or downregulated genes in the 94 knockout strains (from **a** and **b**). Gene set enrichment p-values and fold enrichments were calculated using Fisher's exact test. **d**, Principal component analysis of the 94 knockout strains based on the codon percentages of the genes up- or downregulated at the protein level (genes without mRNA abundance changes). Individual dots are colored in red or blue according to whether they belong to the up- or downregulated gene set as depicted in a and b, respectively. These sets are also emphasized with concentration ellipses depicting normal probability. **e**, Volcano plot of differences in the usage percentage of all 64 codons in the groups of up- or downregulated proteins (genes without mRNA abundance changes) in the 94 knockout strains. Codons enriched in up- or downregulated groups of proteins are depicted in red and blue, respectively. Statistical significance was determined by using two-sided unpaired-samples Student's t-test with a CI of 95%. Violin and boxplots showing the distributions of the two most significantly differentially used codons across the up- and downregulated groups of proteins are inlaid in the graph for illustration. **f**, Distributions of the differences in the percentage of optimal codons between the up- and downregulated genes for ribosomal and non-ribosomal gene knockout strains. The number of strains included in each category is depicted in the plot as n. **b, e and f,** The interquartile range (IQR) is depicted by the box with the median represented by the center line. Whiskers maximally extend to 1.5*IQR. **b and f,** Statistical significance was determined by using two-sided unpaired-samples Wilcoxon rank sum test with a CI of 95%.
