# Supplementary material for: Proteome effects of genome-wide single gene perturbations
Source: Nat Commun. 2022 Oct 18;13:6153. doi: 10.1038/s41467-022-33814-8 (PMC9579165; doi:10.1038/s41467-022-33814-8)
Supplement: Supplementary file 2 — Reporting Summary [file 41467_2022_33814_MOESM2_ESM.pdf]

nature research

Corresponding author(s): Falk Butter
Last updated by author(s): Sep 23, 2022

Reporting Summary

Nature Research wishes to improve the reproducibility of the work that we publish. This form provides structure for consistency and transparency in reporting. For further information on Nature Research policies, see our Editorial Policies and the Editorial Policy Checklist.

Statistics

For all statistical analyses, confirm that the following items are present in the figure legend, table legend, main text, or Methods section.

n/a	Confirmed	
☐	☒	The exact sample size (*n*) for each experimental group/condition, given as a discrete number and unit of measurement
☐	☒	A statement on whether measurements were taken from distinct samples or whether the same sample was measured repeatedly
☐	☒	The statistical test(s) used AND whether they are one- or two-sided<br>*Only common tests should be described solely by name; describe more complex techniques in the Methods section.*
☐	☒	A description of all covariates tested
☐	☒	A description of any assumptions or corrections, such as tests of normality and adjustment for multiple comparisons
☐	☒	A full description of the statistical parameters including central tendency (e.g. means) or other basic estimates (e.g. regression coefficient) AND variation (e.g. standard deviation) or associated estimates of uncertainty (e.g. confidence intervals)
☐	☒	For null hypothesis testing, the test statistic (e.g. *F*, *t*, *r*) with confidence intervals, effect sizes, degrees of freedom and *P* value noted<br>*Give P values as exact values whenever suitable.*
☐	☒	For Bayesian analysis, information on the choice of priors and Markov chain Monte Carlo settings
☐	☒	For hierarchical and complex designs, identification of the appropriate level for tests and full reporting of outcomes
☐	☒	Estimates of effect sizes (e.g. Cohen's *d*, Pearson's *r*), indicating how they were calculated

Our web collection on statistics for biologists contains articles on many of the points above.

Software and code

Policy information about availability of computer code

Data collection	Thermo Xcalibur 3.1
Data analysis	R (3.5.1), NGSpipe2GO with the following modules: FastQC (0.11.8), STAR (2.6.1b), SAMtools (1.5), HTSeq (0.9.0), Subread (1.6.2), BEDTools (2.27.1), Picard (2.17.6), RSeQC (3.0.0), Qualimap (2.2.1), KentUtils (v365), rMATS (4.0.2), FastQScreen (0.12.2), deepTools (3.1.0), BamUtil (1.0.13), and STAR-Fusion (0.8.0), MaxQuant (1.6.1.0), custom algorithms are all deposited in a dedicated github repository https://github.com/m89ozturk/SpombeDeletionLibraryPaper.git

For manuscripts utilizing custom algorithms or software that are central to the research but not yet described in published literature, software must be made available to editors and reviewers. We strongly encourage code deposition in a community repository (e.g. GitHub). See the Nature Research guidelines for submitting code & software for further information.

Data

Policy information about availability of data

All manuscripts must include a data availability statement. This statement should provide the following information, where applicable:

- Accession codes, unique identifiers, or web links for publicly available datasets
- A list of figures that have associated raw data
- A description of any restrictions on data availability

The proteomics data generated in this study have been deposited in the proteomeXchange database under accession code PXD024332 (genome-wide screen) [http://proteomecentral.proteomexchange.org/cgi/GetDataset?ID=PXD024332] and PXD024383 (94 gene set) [http://proteomecentral.proteomexchange.org/cgi/GetDataset?ID=PXD024332]. The transcriptomics data generated in this study have been deposited in the GEO database under accession code GSE167543 (94 gene set) [https://www.ncbi.nlm.nih.gov/geo/query/acc.cgi?acc=GSE167543].

Field-specific reporting

Please select the one below that is the best fit for your research. If you are not sure, read the appropriate sections before making your selection.

☒ Life sciences ☐ Behavioural & social sciences ☐ Ecological, evolutionary & environmental sciences

For a reference copy of the document with all sections, see nature.com/documents/nr-reporting-summary-flat.pdf

Life sciences study design

All studies must disclose on these points even when the disclosure is negative.

Sample size	n=1 for the library determined by do-ability for such a large scale screen. For the 94 strain quantitation quadruplicates were measured for transcriptome and proteome.
Data exclusions	A minimum coverage threshold was set to 1,250 protein groups measured per strain. If not reached, samples were remeasured and the previous measurement excluded.
Replication	94 strains were remeasured in quadruplicates on the proteome. Correlations between these replicates and to the library measurement were calculated. Replicates show good correlations of protein expression values (Pearson's R 0.7 - 0.9).
Randomization	Samples were systematically processed within their 96 plates. However, randomization was predetermined by the provided library where strains had random array positions.
Blinding	The individual gene knockout was blinded as during the experimental stage the gene names were not known and only upon data analysis, the plate positional information were used to identify the knockout strains.

Reporting for specific materials, systems and methods

We require information from authors about some types of materials, experimental systems and methods used in many studies. Here, indicate whether each material, system or method listed is relevant to your study. If you are not sure if a list item applies to your research, read the appropriate section before selecting a response.

Materials & experimental systems

n/a	Involved in the study	
☒	☐	Antibodies
☐	☒	Eukaryotic cell lines
☒	☐	Palaeontology and archaeology
☒	☐	Animals and other organisms
☒	☐	Human research participants
☒	☐	Clinical data
☒	☐	Dual use research of concern

Methods

n/a	Involved in the study	
☒	☐	ChIP-seq
☒	☐	Flow cytometry
☒	☐	MRI-based neuroimaging

Eukaryotic cell lines

Policy information about cell lines

Cell line source(s)	S.pombe knockout library (Bioneer, version M-3030H)
Authentication	commercially provided library
Mycoplasma contamination	not applicable for yeast
Commonly misidentified lines (See ICLAC register)	not applicable
